# Mental and Social Health of Children and Adolescents With Pre-existing Mental or Somatic Problems During the COVID-19 Pandemic Lockdown

**DOI:** 10.3389/fpsyt.2021.692853

**Published:** 2021-07-08

**Authors:** Josjan Zijlmans, Lorynn Teela, Hanneke van Ewijk, Helen Klip, Malindi van der Mheen, Hyun Ruisch, Michiel A. J. Luijten, Maud M. van Muilekom, Kim J. Oostrom, Jan Buitelaar, Pieter J. Hoekstra, Ramón Lindauer, Arne Popma, Wouter Staal, Robert Vermeiren, Hedy A. van Oers, Lotte Haverman, Tinca J. C. Polderman

**Affiliations:** ^1^Department of Child and Adolescent Psychiatry & Psychosocial Care, Amsterdam University Medical Center, Vrije Universiteit Amsterdam, Amsterdam Public Health, Amsterdam, Netherlands; ^2^Emma Children's Hospital, Amsterdam University Medical Center, Child and Adolescent Psychiatry & Psychosocial Care, Amsterdam Reproduction and Development, Amsterdam Public Health, University of Amsterdam, Amsterdam, Netherlands; ^3^Curium-Leiden University Medical Center, Leiden University Medical Center, Leiden, Netherlands; ^4^Karakter Child and Adolescent Psychiatry University Centre, Nijmegen, Netherlands; ^5^Department of Child and Adolescent Psychiatry, Amsterdam University Medical Center, University of Amsterdam, Amsterdam, Netherlands; ^6^Levvel, Academic Center for Child and Adolescent Psychiatry, Amsterdam, Netherlands; ^7^Department of Child and Adolescent Psychiatry, University Medical Center Groningen, University of Groningen, Groningen, Netherlands; ^8^Amsterdam University Medical Center, Vrije Universiteit Amsterdam, Epidemiology and Data Science, Amsterdam Public Health, Amsterdam, Netherlands; ^9^Department of Cognitive Neuroscience, Donders Institute for Brain, Cognition and Behaviour, Radboudumc, Nijmegen, Netherlands

**Keywords:** mental health, COVID-19, child and adolescent psychiatry, child self-report, depression, anxiety, sleep-related impairment, peer relations

## Abstract

**Background:** The COVID-19 lockdown increases psychological problems in children and adolescents from the general population. Here we investigate the mental and social health during the COVID-19 lockdown in children and adolescents with pre-existing mental or somatic problems.

**Methods:** We included participants (8–18 years) from a psychiatric (*N* = 249) and pediatric (*N* = 90) sample, and compared them to a general population sample (*N* = 844). Measures were assessed during the first lockdown (April-May 2020) in the Netherlands. Main outcome measures were Patient-Reported Outcomes Measurement Information System (PROMIS®) domains: Global Health, Peer Relationships, Anxiety, Depressive Symptoms, Anger, and Sleep-Related Impairment, as reported by children and youth. Additionally, socio-demographic variables, COVID-19-related questions, changes in atmosphere at home from a parent and child perspective, and children's experiences of lockdown regulations were reported by parents.

**Results:** On all measures except Global Health, the pediatric sample reported least problems. The psychiatric sample reported significantly more problems than the general population sample on all measures except for Anxiety and Peer Relationships. Having a COVID-19 affected friend/relative and a COVID-19 related change in parental work situation negatively moderated outcome, but not in the samples with pre-existing problems. All parents reported significant decreases in atmosphere at home, as did children from the general population.

**Conclusion:** We observed significant differences in mental and social health between three child and adolescent samples during the COVID-19 pandemic lockdown and identified COVID-19-related factors influencing mental and social health.

## Background

It is well-known that large scale disasters have great impact on the well-being of the general population. Whether human-induced (e.g., the World Trade Center attacks), natural (e.g., hurricanes), or technological (e.g., Chernobyl nuclear reactor accident), disasters are accompanied by an increase in a wide scale of mental and behavioral disorders (1). Pandemics specifically can lead to increased levels of anxiety, depression, and post-traumatic stress (2, 3). The coronavirus disease 2019 (COVID-19) pandemic is a major game changer in the life of many individuals across the world. Important direct consequences that highly affect social life are regulations imposed by governments, such as closing schools, and leisure and sports facilities, and obligating social distancing in the public space. During COVID-19, increased rates of anxiety and depression, and decreased psychological well-being have been reported in the adult general population (4), and adult psychiatric patients show worsening of their symptoms (5).

Empirical studies on mental and social health during the COVID-19 pandemic in children and adolescents are limited (6), though several cross-sectional Chinese studies have been published. These studies have shown that 20–40% of Chinese children and adolescents reported psychological problems during the COVID-19 pandemic, especially symptoms of anxiety, depression, and post-traumatic stress (7–9). Very recently, a Dutch study similarly showed that the mental and social health of children and adolescents from the general population had deteriorated since the COVID-19 lockdown (10). Growing up in a single-parent family, having more than three children in the family, a negative change in parental work situation due to COVID-19 regulations, and having a friend or relative infected with COVID-19 were factors associated with more mental and social health problems during the COVID-19 lockdown.

The current study extends these efforts by investigating mental and social health in vulnerable populations, namely children and adolescents with pre-existing mental problems or with a (chronic) somatic condition, during the first Dutch COVID-19 lockdown. During this lockdown, schools, leisure facilities, bars, and restaurants were closed, people were strongly advised to work from home as much as possible and to comply to social distancing, and it was not allowed to be outside with more than two people unless they were from the same household.

Children with mental problems and chronic somatic conditions are known to be at risk for psychosocial problems. In general, they rate their well-being, emotional and social functioning, and psychosocial health lower than their healthy peers (11–13). The extent to which these children experience a lower mental and social health, however, varies per condition (13, 14). For instance, the level of psychopathology, degree of stressful events, gender, and school attendance are associated with psychosocial functioning (15, 16). Therefore, it is important to examine mental and social health during the COVID-19 lockdown in which many of these factors are involved (17, 18). Also, psychosocial problems may arise or worsen because of the disruption of (mental) support programs due to COVID-19 regulations (19). In addition, treatments of children and their families suddenly transitioned from in-person therapy to online therapy, which may in some cases affect the quality of their treatment (20). To date, empirical studies of the mental and social health of these vulnerable populations during the COVID-19 lockdown are still sparse.

As of yet, few empirical studies have investigated the mental and social effects of COVID-19 related regulations in children with pre-existing mental problems (psychiatric population) and results are mixed. One study in children with autism spectrum disorder (ASD) reported increases in irritability, hyperactivity, inappropriate speech, and speech quality from before to during the pandemic (21). Another study in a neuropsychiatric child and adolescent sample reported increases in obsessive-compulsive problems, post-traumatic stress, and thought problems (22). On the other hand, a French study investigated the well-being of 533 children and adolescents with self-reported Attention Deficit/Hyperactivity Disorder (ADHD) during the COVID-19 pandemic (23). As reported by their parents, 65.3% of these children showed no changes in well-being or were doing better than before the pandemic, suggesting that more than half of the children and adolescents with ADHD experience stability or improvement of their well-being during the COVID-19 lockdown. Still, one third of children reported lower well-being, indicating substantial variation in responses. Similarly, anecdotal evidence from child and adolescent clinical practice suggested that for some children and families, COVID-19 lockdown-induced changes reduced sensory exposure and daily stress (e.g., school related stress), and subsequently seemed to reduce mental problems and even improved well-being in some children, while for other children negative outcomes (e.g., increased stress levels, reduced well-being and mental health) are to be expected (24).

The literature on the consequences of the COVID-19 lockdown in children with a (chronic) somatic condition (pediatric population) is also limited. A recent study has shown that psychosocial stress in children and adolescents with cancer did not increase during the first months of the COVID-19 pandemic in the Netherlands (25). Additionally, adolescents with cystic fibrosis reported lower anxiety levels during the COVID-19 pandemic compared to their healthy peers (26). These studies suggest that children with a (chronic) somatic condition, despite their vulnerability, may be less susceptible to negative consequences of the COVID-19 pandemic. However, a recent study in a large Hongkong population (N 29,202 families) showed that children with chronic diseases and children with parents reporting mental problems scored higher on emotional and behavioral problems, and decreased peer relations (27). In sum, research in vulnerable groups of children and adolescents during the COVID-19 pandemic is currently scarce, and findings hint at positive as well as negative consequences on mental and social health.

The aim of the current study was to assess the mental and social health of a broad population of children with pre-existing conditions (psychiatric or pediatric) during the first COVID-19 lockdown in the Netherlands and to compare their global health, peer relationships, symptom levels of anxiety, depression and anger, and sleep-related impairment to the general population of children and adolescents. We examined potential associated factors, including COVID-19 specific factors such as having an affected friend or relative. In addition, we described changes in home atmosphere due to the first Dutch COVID-19 lockdown and report qualitative data on children's experiences of the COVID-19 regulations.

## Methods

### The First COVID-19 Lockdown in the Netherlands

On March 15th 2020, the first COVID-19 lockdown came into practice in the Netherlands. From that moment on, all schools and child day-care facilities were closed (unless one or both parents had a profession that was classified as essential), as well as hairdressers, sport and leisure facilities, bars, and restaurants. Adults were advised to work from home as much as possible. People over the age of 12 were advised to keep social distance (1.5 m), to take hygiene measures (e.g., washing hands, use of tissue papers, sneezing in elbow), and traveling was discouraged. However, it was still permitted to go outside by oneself, to receive three guests at home, and young children (<12 years) could play outside with their friends. From April 28th 2020, young children (<12 years) were again allowed to play sports outside in a team. On May 11th 2020, were partially reopened, followed by the secondary schools on June 2nd 2020.

### Procedure

All families were invited to participate during the first COVID-19 lockdown in The Netherlands (between the end of April and early May 2020). During our measurement window, no changes (except for team sports for children <12 years) in Dutch COVID-19 regulations occurred. Parents were approached via email and children were subsequently approached by their parents. If parents and children were willing to participate, the parents completed a sociodemographic questionnaire and COVID-19-related questions online. The children also completed online COVID-19-related questions and several validated Patient-Reported Outcome Measures (PROMs). We collected data through research websites of the KLIK PROM portal developed specifically for each group. The completion time for children and parents together was ~15 min. All children and parents provided informed consent and the study was approved by the Medical Ethics Committee of the Amsterdam UMC.

### Participants

We included independent samples of children and adolescents (8–18 years) from child and adolescent psychiatric centers and a children's hospital and compared these to a sample from the general population.

The psychiatric sample consists of children receiving psychiatric care for varying problems (e.g., autism, depression, ADHD) at one of four tertiary academic child and adolescent psychiatric centers in the Netherlands: Accare, Levvel, Curium, and Karakter. The four centers cover child and adolescent psychiatric care in the northern, western, and eastern part of the Netherlands. We approached parents through email between April 30th and May 12th, 2020. In total, we invited 5,615 children and their parents to participate (response rate 4.4%).

The pediatric sample consists of children with a (chronic) somatic condition (e.g., juvenile idiopathic arthritis, endocrinological diseases, cystic fibrosis) who are under treatment in the Emma Children's Hospital Amsterdam UMC. Children who already complete PROMs as standard part of their care were included. We approached parents via email in the first week of May 2020. In total, we invited 1,150 children and their parents to participate (response rate 7.3%).

For the general population sample, data were collected via an independent online research agency “Panel Inzicht” in April 2020 [see (10) for additional information on the general population sample]. Parents were invited until 1,000 responses were reached (response rate 8.4%).

### Socio-Demographic and COVID-Related Measures

The socio-demographic questionnaire was completed by parents and comprised questions about themselves (region, country of birth, education level, marital status, and number of children) and their child (age, gender). The COVID-19-related questions for parents concerned whether the parent or partner reported a negative change in parental work situation due to COVID-19 (i.e., loss of income, reduced number of working hours, unemployment) and whether or not a friend or relative was infected with COVID-19. Additionally, parents completed two questions regarding the atmosphere at home before and during the first Dutch COVID-19 lockdown on a visual analog scale (range 0–100): “How did you experience the atmosphere at home before the Corona regulations?” and “How do you experience the atmosphere at home now?”

The COVID-19-related questions for children also assessed the atmosphere at home: “How did you experience atmosphere at home before the schools were closed?” and “How do you experience the atmosphere at home now?” Finally, we asked an open-ended question: “How are the Corona-regulations for you?”

### Mental and Social Health Outcomes

We administered six Dutch Patient-Reported Outcomes Measurement Information System (PROMIS®) pediatric measures (8–18 years) to children and youth: Scale V1.0—Global health (e.g., In general, how would you rate your mental health, including your mood and your ability to think?) (28), CAT V2.0—Peer Relationships (e.g., I could talk with my friends) (29), CAT V2.0—Anxiety (e.g., I worried about what could happen to me) (30), CAT V2.0—Depressive Symptoms (e.g., I felt sad) (30), Scale V2.0—Anger (e.g., I was so angry I felt like throwing something) (31), and CAT V1.0—Sleep-related Impairment (e.g., I was sleepy during the daytime) (32). These measures have been selected by the American Psychiatric Association (APA) as level-2 assessment measures for monitoring and evaluating psychiatric disorders from the DSM-V. The psychometric properties of the PROMIS measures are adequate. Previous studies have shown that the instruments provide reliable measurements at the mean of the population and at least two standard deviations in clinically relevant direction (28–32). PROMIS measures use a 7-days recall period and items are scored on a five-point Likert scale. All items range from “never” to “(almost) always”, except for Global Health, where response categories differ for each item (e.g., “excellent” to “poor”). Total scores are calculated by transforming item scores into a T-score ranging from 0 to 100, with a mean of 50 and a standard deviation of 10 in the U.S. general population (for our purposes, we compared to representative Dutch data; see data analysis). For all measures, higher scores represent more of the construct. For Anger (nine items) and Global Health (7+2 items) we used the total scales. For the other item banks, we used Computerized Adaptive Testing (CAT), in which items are presented based on the responses to previously administered items. This results in a reliable total score while administering fewer items than regular questionnaires (33).

### Data Analysis

We used the Statistical Package for Social Sciences (SPSS) version 26.0 for statistical analyses. First, we characterized participants of each sample using descriptive analysis (means and percentages).

Second, we performed analyses of covariance (ANCOVA) to assess differences in PROMIS outcome measures between samples. We corrected for age, sex, and parental country of birth as these are characteristics known to influence mental and social outcomes (11, 34, 35). We performed Bonferroni-*corrected post-hoc t*-tests to determine which samples differed significantly from each other. Next, we tested whether there were differences in proportions of children reporting severe symptoms or poor functioning between samples. “Severe” symptoms and “poor” functioning were defined as 1.5 SD above or below the mean T-score of an independent and representative pre-COVID-19 Dutch sample (36, 37), except for Peer Relations, where 2 SD was used as the cut-off value (see www.HealthMeasures.net).

Third, to determine which variables were associated with the PROMIS measures during COVID-19, we repeated the ANCOVA over all groups and included pertinent independent variables: marital status, region, number of children in the family, parental educational level, change in parental work situation due to COVID-19, and infected relative/friend with COVID-19. As the proportion of the participants from the general population is high, we repeated the ANCOVA without the general population sample to explore whether effects are mainly driven by this group.

Fourth, we performed paired samples *T*-tests in each group to investigate changes in atmosphere at home from before to during COVID-19 as reported by children and parents. In addition, we performed an ANCOVA to assess whether the changes in atmosphere differed between samples, correcting for age and sex.

Finally, two authors qualitatively analyzed the children's answers to the open-ended question “How are the corona-regulations for you?” using the method for thematic analysis in psychology (38). Answers were categorized into positive, neutral, or negative experiences and clustered into main themes. Themes were ranked based on the number of times mentioned by children (most to least mentioned). We analyzed answers from all groups together but explored differences between groups.

## Results

### Sociodemographic Characteristics

Sociodemographic characteristics of all samples are presented in Table 1. In the psychiatric sample 249 children participated (response rate: 4.4%). The mean age was 12.8 years, 56.2% was male, 17.7% had an infected relative/friend, and 18.9% of their parents experienced a negative change in parental work situation due to COVID-19.

**Table 1:** Socio-demographic characteristics of participants per group.

	**Child/adolescent psychiatric sample**	**Pediatric sample**	**General population sample**
*N*	249	90	844
Mean age in years (SD) (range)	12.8 (2.9) (8–18)	12.9 (3.3) (8–18)	13.4 (2.8) (8–18)
	%	%	%
Sex (male)	56.2	55.6	47.4
**Region^∧^**			
North	30.1	3.3	12.6
East	26.1	13.3	22.2
South	1.2	1.1	25.0
West	42.6	82.2	40.3
**Number of children in family**			
1 child	16.9	18.2	25.5
2 children	53.4	51.1	46.6
3 children or more	29.7	30.7	27.9
**Country of birth parents**			
Netherlands (both parents)	83.9	83.1	88.2
Foreign country (at least one parent)	16.1	16.9	11.8
**Marital status parents**			
Two-parent family	76.8	81.1	82.0
Single-parent family	23.2	18.9	18.0
**Educational level parents°**			
Low	9.2	6.7	9.0
Intermediate	43.8	45.6	51.8
High	47.0	47.8	39.2
**COVID-19-specific variables**			
Infected relative/friend (yes)	17.7	33.3	23.7
Negative change in work situation (yes)	18.9	21.1	26.2

*Due to missing values, number of respondents differs slightly across socio-demographic variables, minimal Ns are 815, 243, and 88*.

∧ *Region: North = Groningen, Friesland, Drenthe, East = Overijssel, Gelderland, Flevoland, South = Zeeland, Noord-Brabant, Limburg, West = Utrecht, Noord-Holland, Zuid-Holland*.

° *Educational level parents, Low; primary, lower vocational education, lower and middle general secondary education; Intermediate: middle vocational education, higher secondary education, pre-university education; High: higher vocational education, university*.

In the pediatric sample 90 children participated (response rate: 7.3%). The mean age was 12.9 years, 55.6% was male, 33.3% had an infected relative/friend, and 21.1% of their parents had a negative change in parental work situation due to COVID-19.

In the general population sample 844 children participated (response rate: 8.4%). The mean age was 13.4 years, 47.4% was male, 23.7% had an infected relative/friend, and 26.2% of their parents experienced a negative change in parental work situation due to COVID-19.

### Between Group Differences in Mental and Social Health

We found significant differences on all self-reported PROMIS measures between the three groups (*p* < 0.01, η^2^ = 0.03–0.04) after controlling for the covariates age, sex, and parental country of birth (see Table 2). The psychiatric sample reported worst mental and social health and the pediatric sample reported best mental and social health, whereas the general population had scores in between. *Post-hoc t*-tests revealed that the psychiatric sample reported significantly worse scores than the general population (*p* < 0.05) on the Global Health (ΔM = −3.31, CI = −4.58; −2.05), Depressive Symptoms (ΔM = 2.03, CI = 0.51; 3.56), Anger (ΔM = 3.24, CI = 1.75; 4.74), and Sleep-Related Impairment (ΔM = 2.97, CI = 1.41; 4.53) measures.

**Table 2:** Mean PROMIS T-scores in the different samples during COVID-19, corrected by age, sex, and parental country of birth.

	**Child/adolescent psychiatric sample (1)** ***N =*** **242**		**Pediatric sample (2)** ***N =*** **89**		**General population sample (3)** ***N =*** **813**		p^*^	*****η^2^*****
	***M***	***SD***	***M***	***SD***	***M***	***SD***		
PROMIS global health^**+**^	43.0^b,c^	8.0	46.5^c^	7.6	46.2^a^	6.9	<0.01	0.04
PROMIS peer relationships^**+**^	43.5^b^	8.9	49.3^a,c^	8.2	44.3^b^	7.0	<0.01	0.04
PROMIS anxiety°	51.3^b^	8.8	45.0^a,c^	8.0	50.5^b^	7.6	<0.01	0.04
PROMIS depressive symptoms°	51.5^b,c^	10.4	45.2^a,c^	8.0	49.4^a,b^	8.0	<0.01	0.03
PROMIS anger°	50.9^b,c^	9.6	43.8^a,c^	8.0	47.3^a,b^	8.2	<0.01	0.04
PROMIS sleep related impairment°	52.8^a,c^	9.4	47.5^a,c^	8.3	49.9^a,b^	8.7	<0.01	0.03

* *P-value of the main effect of the ANCOVA*.

+ *Higher scores indicate better functioning*.

° *Higher scores indicate more symptoms*.

a,b,c *Represent significant differences (p < 0.05, Bonferroni corrected) between samples as indicated by post-hoc Tukey tests*.

The pediatric sample reported significantly better scores than the general population sample (*p* < 0.05) on Peer Relationships (ΔM = 5.09, CI = 3.06; 7.12), Anxiety (ΔM = −5.67, CI = −7.79; −3.55), Depressive Symptoms (ΔM = −4.23, CI = −6.52; −1.94), Anger (ΔM = −3.72, CI = −5.97;−1.46), and Sleep-Related Impairment (ΔM = −2.44; CI = −4.79; −0.08).

The psychiatric sample reported significantly worse scores than the pediatric sample (*p* < 0.05) on all six PROMIS domains: Global Health (ΔM = −3.53, CI = −5.62; −1.39), Peer Relationships (ΔM = −5.87, CI = −8.12; −3.62), Anxiety (ΔM = 6.20, CI = 3.86; 8.55), Depressive Symptoms (ΔM = 6.26; CI = 3.72; 8.80), Anger (ΔM = 6.96, CI = 4.46; 9.45), and Sleep-Related Impairment (ΔM = 5.40, CI = 2.80; 8.01).

**Figure 1:**
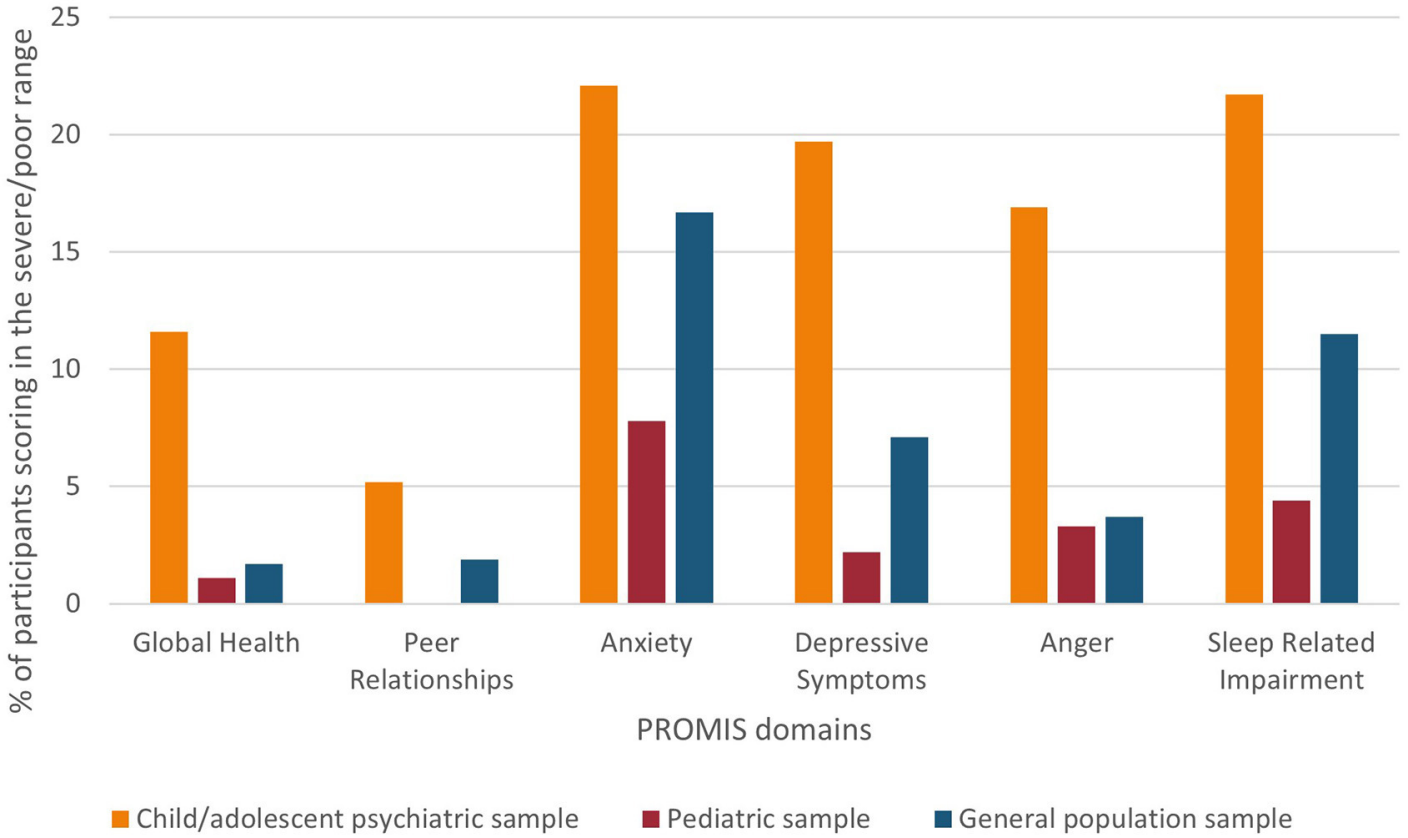
Percentage of participants scoring in the severe/poor range (>1.5 SD)* on PROMIS domains for all samples. *For Peer Relationships a cut-off of 2 SD was used, following the Health Measures guidelines.

Likewise, the percentages of severe/poor scores were highest in the psychiatric sample (range = 5.2–22.1%) and lowest in the pediatric sample (range = 0–7.8%; see Figure 1).

### Variables Associated With Mental and Social Health During COVID-19

In Table 3, we report the ANCOVA regression coefficients of the independent variables including all three groups. Higher age was significantly associated (*p* < 0.01) with lower Global Health (b = −0.21, CI = −0.35; −0.06), higher Anxiety (b = −0.28, CI = −0.44; −0.11), and lower Anger (b = −0.49, CI = −0.66; −0.32). Male gender was significantly associated with higher Global Health (b = 1.19, CI = 0.36; 2.02), lower Peer Relationships (b = −1.11, CI = −2.00; −0.22), lower Anxiety (b = −1.04, CI = −1.95; −0.12), lower Depressive Symptoms (b = −1.55, CI = −2.55; −0.55), and lower Sleep–related Impairment (b = −1.37; CI = −2.39; −0.35). Living in the northern region of the Netherlands was significantly (*p* < 0.05) associated with lower scores on Depressive Symptoms (b = −2.00; CI = −3.51; 0.48). Being from a single–parent family was significantly associated (*p* < 0.05) with lower Global health (b = −2.14, CI = −3.21; −1.08) and higher Anxiety (b = 1.30, CI = 0.13; 2.48). Having three children or more within the household was significantly associated (*p* < 0.05) with higher Anger (b = 1.77, CI = 0.38; 3.15). For each outcome measure, the group effect remained.

**Table 3:** Unstandardized regression coefficients of independent variables of the ANCOVA for mental and social health outcomes.

	**Global health**	**Peer relationships**	**Anxiety**	**Depressive symptoms**	**Anger**	**Sleep-Related Impairment**
Predictors	B	B	B	B	B	B
Age	−0.21^**^	0.02	−0.28^*^	−0.04	−0.49^**^	0.00
Male sex	1.20^*^	−1.11^*^	−1.04^*^	−1.55^*^	−0.12	−1.37^**^
**Region**^**∧**^						
West	Ref	Ref	Ref	Ref	Ref	Ref
North	0.26	0.65	−1.28	−2.00^*^	−1.36	−0.84
East	−0.21	0.06	0.67	−0.08	0.08	0.84
South	−0.61	−0.07	−0.00	−0.68	−0.61	−0.68
Foreign country of birth parents	−0.97	0.07	0.52	0.40	0.29	1.35
**Educational level parents°**						
Low	Ref	Ref	Ref	Ref	Ref	Ref
Intermediate	−0.50	0.62	1.17	2.02	1.88	1.64
High	0.73	1.00	1.02	1.84	1.71	0.63
Single–parent family	−2.14^**^	−1.09	1.30^*^	0.74	0.33	1.31
**Number of children in family**						
1 child	Ref	Ref	Ref	Ref	Ref	Ref
2 children	0.08	−0.69	−0.17	−0.03	0.93	0.31
3 or more children	0.81	0.15	−0.34	−0.02	1.77^*^	0.48
Negative change in work situation	−0.47	−0.22	2.11^**^	1.72^**^	1.17	2.01^**^
Infected relative/friend with COVID−19	0.74	0.02	1.44^**^	0.97	−0.12	0.74

*B = Unstandardized regression coefficient of multivariable linear regression model*.

** *p < 0.01*,

* *p < 0.05*.

∧ *Region: North = Groningen, Friesland, Drenthe, East = Overijssel, Gelderland, Flevoland, South = Zeeland, Noord-Brabant, Limburg, West = Utrecht, Noord-Holland, Zuid-Holland*.

° *Educational level parents divided into three categories: Low = primary, lower vocational education, lower and middle general secondary education; Intermediate = middle vocational education, higher secondary education, pre-university education; High = higher vocational education, university*.

Regarding COVID-19-specific variables, a negative change in parental work situation was significantly associated (*p* < 0.01) with more Anxiety (b = 2.11; CI = 1.05; 3.18), more Depressive Symptoms (b = 1.72, CI = 0.56; 2.88), more Anger (b = 1.17, CI = 0.03; 2.31), and more Sleep-Related Impairment (b = 2.01, CI = 0.82; 3.20). A parent having a friend or relative infected with COVID-19 was significantly associated (*p* < 0.01) with a higher Anxiety score (b = 1.44, CI = 0.35; 2.53).

Finally, we performed the ANCOVA analyses again in the psychiatric and pediatric groups separately to explore whether effects are driven mainly by the general population sample. In the psychiatric group, we found higher age to be significantly associated (*p* < 0.05) with lower Global Health (b = −0.58, CI = −0.95; −0.22) and lower Anger (b = −0.66, CI = −1.11; −0.21). Male gender was significantly associated (*p* < 0.05) with higher Global Health (b = 2.66, CI = 0.57; 4.75), and lower Depressive Symptoms (b = −2.83, CI = −5.64; −0.02). We found no associations between COVID-19-specific variables and the social and mental health outcomes in the psychiatric group.

In the pediatric group, we found higher age to be significantly associated (*p* < 0.05) with higher Sleep-Related Impairment (b = 0.63, CI = 0.02; 1.24). Male gender was significantly associated (*p* < 0.05) with lower Depressive Symptoms (b = −4.45, CI = −7.87; −0.94). A parent having a friend or relative infected with COVID-19 was significantly associated (*p* < 0.05) with lower Peer Relationships (b = −4.57, CI = −8.62; −0.52).

### Atmosphere at Home Before and During COVID-19

Children from the psychiatric and pediatric samples reported no differences in atmosphere at home between before (measured retrospectively) and during the COVID-19 lockdown, whereas children in the general population reported a worse atmosphere (ΔM = −3.13, *p* < 0.001) at home during the COVID-19 lockdown. All parents reported a negative change in atmosphere (psychiatric sample ΔM = −2.76, pediatric sample ΔM = −3.38, and general population sample ΔM = −4.62; all ps <0.05; see Table 4). Furthermore, we investigated whether changes in atmosphere differed between the samples. The ANCOVA indicated no significant differences between the samples for children (*p* = 0.055, η^2^ = 0.005) and parents (*p* = 0.062, η^2^ = 0.005), corrected for age and gender.

**Table 4:** Atmosphere at home as experienced by children and parents.

	**Atmosphere before COVID-19**	**Atmosphere during COVID-19**	**Change in Atmosphere**	P^*^
	***M***	***M***	***M***	
**Child/Adolescent psychiatric sample (*****N =*** **249)**				
Children	72.9	72.4	−0.6	0.675
Parents	74.1	71.4	−2.8	**0.013**
**Pediatric sample (*****N =*** **90)**				
Children	86.3	84.3	−2.0	0.241
Parents	87.1	83.8	−3.4	**0.028**
**General population sample (*****N =*** **844)**				
Children	81.4	78.2	−3.1	**<0.001**
Parents	81.0	76.4	−4.6	**<0.001**

* *P-value of the paired sample T-test. A p < 0.05 indicates significant differences, shown in bold. Parents answered the questions “How did you experience the atmosphere at home before the Corona regulations?” and “How do you experience the atmosphere at home now?” Children answered the questions: “How did you experience atmosphere at home before the schools were closed?” and “How do you experience the atmosphere at home now?” Answers were given on a visual analog scale (range 0–100)*.

### Impact of the COVID-19-Regulations on Daily Life

In general, children and adolescents from the vulnerable samples reported similar experiences with the COVID-19-regulations as the general population. Most children (86–92%) indicated that the COVID-19-regulations negatively impacted their daily life. The main problems they experienced (≥5% of the children per group) were missing contact with friends (~42%), not being allowed to go to school (~22%), missing freedom (~16%), not being allowed to participate in sports (~14%), missing joyful activities such as birthdays, shopping, day trips, and (graduation) parties (~13%), and missing extended family (especially grandparents; ~8%). However, there were some small differences between the experiences of the samples. Only children and adolescents from the pediatric and psychiatric sample reported difficulties with keeping social distance (e.g., hard to keep distance in daily life, not allowed to hug, and getting warnings from other people), whereas the general population sample was the only group that mentioned boredom due to the COVID-19-regulations. In addition, only children from the psychiatric sample indicated that the (continually changing) COVID-19-measures made them angry, sad, or insecure. A minority of children reported that their daily life was not affected by COVID-19-regulations (5–11%) or reported that the COVID-19-regulations positively influenced their daily life (e.g., “I actually like the measures, less social contact and clear rules,” “I like to be at home”; 3–5%). See Table 5 for an overview.

**Table 5:** Overview of the most mentioned themes (≥5% of the children per sample) in response to the open question “How are the corona-regulations for you?” per sample, ranked by the number of times mentioned (most to least).

**Child/adolescent psychiatric sample (*N* = 249)**	**Pediatric sample (*N* = 90)**	**General population sample (*N* = 844)**
1. Missing contact with friends “I have not seen any of my friends in 7 weeks”	1. Missing contact with friends “I miss my friends the most, because I cannot meet them right now”	1. Missing contact with friends “Feeling alone, because I cannot see my friends anymore, it is different online”
2. Not allowed to go to school “It is a pity that I cannot go to school”	2. Not allowed to go to school “I would like to go back to school”	2. Not allowed to go to school “I miss my teacher and my class”
3. Missing freedom “I am almost not allowed to go outside and therefore I cannot lose my energy”	3. Missing freedom “That I am not allowed to go anywhere anymore makes me feel trapped”	3. Missing freedom “I am only allowed to play in and around the house”
4. Missing joyful activities “I am not allowed to go to the pool or a day out”	4. Not allowed to participate in sports “I miss sports”	4. Not allowed to participate in sports “I used to play soccer three times a week, I miss playing sports and being outside the most”
5. No problems with corona-regulations “I am not bothered by the corona-regulations, I like that I get more rest”	5. Missing joyful activities “Birthday parties are canceled, I miss doing fun things together”	5. Missing joyful activities “I want to party with my friends, to participate in my final exams and I want to go on a holiday but that is not possible now”
6. Not allowed to participate in sports “My judo training is canceled and I find that very unfortunate”	6. Missing extended family “I am not allowed to visit my grandparents”	6. Difficulties with homeschooling “I miss my daily routine and the boundary between school and home is completely gone”
7. Difficulties with homeschooling “Homeschooling is difficult, especially having my parents as teachers”	**7. Difficulty keeping distance** “People tell me that I am not allowed to stand too close to my friends”	7. Missing extended family “I am not allowed to see or hug my grandparents”
**8. Difficulty keeping distance** “Keeping distance from family (not hugging) and keeping distance from other people is difficult”	8. No problems with corona-regulations “I am not bothered by the corona-regulations”	**8. Boredom** “I am often bored, because we cannot do anything”
9. Missing extended family “I miss my extended family”		9. No problems with corona-regulations “It does not bother me”
**10. Emotions about regulations** “I am not allowed to do everything I want, I do not like that and it makes me angry”		

*White: 5–15, Light gray: 16–30, Dark gray: 31–50%. Themes represented in bold are not mentioned by all groups*.

## Discussion

In this study, we compared the mental and social health during the first Dutch COVID-19 pandemic lockdown in the spring of 2020 of children and adolescents with pre-existing psychiatric and somatic problems to a general population sample. We assessed global health, peer relationships, anxiety, depressive symptoms, anger, and sleep-related impairment. Mental and social health were worst for children with pre-existing psychiatric problems, whereas children from the pediatric sample showed the most favorable results.

As expected, children with pre-existing psychiatric problems showed most problems in mental and social health. The closing of schools and other services during the COVID-19 pandemic lockdown likely affects this group of children especially since these are places where vulnerable children often seek help first (39). Furthermore, access to professional mental health care substantially changed during the COVID-19 pandemic lockdown. Mental health care facilities were partly disrupted and had to rapidly adapt their services, while being perceived as having a higher threshold for help seeking, due to fear of infection. Additionally, people may have felt that mental problems are less important when the overall medical system is under high pressure because of COVID-19. Personal contact often changed to online therapy, of which the effects have not yet been tested thoroughly (40). Although online therapy can be an effective treatment, during the pandemic online therapy is sometimes provided out of necessity rather than choice. In addition, online therapy might increase the risk of losing contact with particular vulnerable families (20). Furthermore, existing feelings of anxiety might increase in response to the threat of close relatives being infected with COVID-19 or becoming infected oneself. The finding that children with pre-existing problems living in the north of the Netherlands showed the least anxiety problems might hint to this, since in that part of the country almost no reported COVID-19 cases were present at the time. Still, these interpretations are made with caution since based on the current data we cannot conclude that the COVID-19 lockdown plays a significant role in the higher levels of mental and social problems in the psychiatric population. The only data that clearly hint at this notion derive from the qualitative part of our study showing that only children from the psychiatric sample indicated that the (continually changing) COVID-19-measures made them angry, sad, or insecure.

In contrast to the psychiatric sample, children, and adolescents from the pediatric sample showed the least problems on all mental and social health scales. As this population generally suffers from more psychosocial problems than the general population (11, 12), this suggests they are least negatively affected by the COVID-19 pandemic lockdown regulations. A possible explanation for this finding may be that these children are more often confronted with stressful events that directly affect their physical health and restraints in daily life due to the management of their disease. As a result, children may have developed more adaptive coping strategies (25, 26). In addition, one could speculate that the COVID-19 pandemic lockdown induced changes in daily life might be less invasive for some of these children as home schooling and carefully taking care of one's physical health may be part of life already (25). Likewise, they may be more used to having online contact with friends, which may explain their higher reported Peer Relationships.

Concerning COVID-19-specific variables, a negative change in parental work situation and having an infected friend or relative contributed to various mental and social health problems (i.e., Anxiety, Depressive Symptoms, Anger, and Sleep Related Impairment) in the general population [see (10) for results in the general population specifically], but not in our sample of children and adolescents with pre-existing mental problems. In our sample of children and adolescents with pre-existing somatic problems, we only found a small negative effect of a parent having a friend or relative infected with COVID-19 on the child's peer relations. It is possible that these vulnerable children are less affected by such situations because they are more accustomed to dealing with stressful events and thereby have become more resilient to them. Since the qualitative part of this study suggested that the COVID-19-restrictions itself do affect mental health in the psychiatric population, it is important to examine additional COVID-19 related measures in the future.

Limitations of our study include low response rates and a potential selection bias of our samples, which limit extrapolation of our results. In particular, the pediatric sample was relatively small. Additionally, due to the cross-sectional design of this study, we cannot assess what factors have impacted on the *change* in mental and social health of children and adolescents due to the COVID-19 pandemic lockdown. Finally, there may be individual differences in how children respond to the lockdown. The literature so far hints at a potential diverse range of effects that the COVID-19 pandemic has on children and adolescents with pre-existing vulnerabilities, ranging from an increase in mental health problems to reduction of stress, loss of social pressure, and improved well-being (24). For future research, we recommend studying this variability more in depth.

This study contributes to “a call for action for mental health science” by Holmes et al. (41) who argue that this type of research should be one of the main priorities in research during the COVID-19 pandemic. Our findings present descriptive data that compare mental and social health between vulnerable groups of children and adolescents and a general population. Future studies need to examine additional factors related to the COVID-19 pandemic lockdown and should apply longitudinal designs which will enable tracking the course of mental and social health when the COVID-19 lockdown regulations continue or return in the future.

## Data Availability Statement

The raw data supporting the conclusions of this article will be made available by the authors, without undue reservation.

## Ethics Statement

The studies involving human participants were reviewed and approved by The Medical Ethical Committee of the Amsterdam UMC. Written informed consent to participate in this study was provided both by the participants and their legal guardians/next of kin.

## Author Contributions

JZ, LT, KO, AP, HO, LH, and TP designed the study. JZ, LT, HE, HK, MMh, HR, ML, and MMu acquired, processed, or analyzed the data. JZ, LT, and TP drafted the manuscript. All authors have worked on the revision on the manuscript, have approved the submitted version (and any substantially modified version that involves the author's contribution to the study), have agreed both to be personally accountable for the author's own contributions and to ensure that questions related to the accuracy or integrity of any part of the work, even ones in which the author was not personally involved, are appropriately investigated, resolved, and the resolution documented in the literature.

## Conflict of Interest

JB has served as a member of the advisory board of Medice, Roche, Servier, Angelini and has received speaker fee from Takeda and Medice. The remaining authors declare that the research was conducted in the absence of any commercial or financial relationships that could be construed as a potential conflict of interest.

## References

[1] Neria Y Nandi A Galea S. Post-traumatic stress disorder following disasters: a systematic review. Psychol Med. (2008) 38:467–80. 10.1017/S0033291707001353 17803838 PMC4877688

[2] Jalloh MF Li W Bunnell RE Ethier KA O'Leary A Hageman KM. Impact of Ebola experiences and risk perceptions on mental health in Sierra Leone, July 2015. BMJ Glob Heal. (2018) 3:1–1. 10.1136/bmjgh-2017-000471 29607096 PMC5873549

[3] Sprang G Silman M. Posttraumatic stress disorder in parents and youth after health-related disasters. Disaster Med Public Health Prep. (2013) 7:105–10. 10.1017/dmp.2013.22 24618142

[4] Kwong ASF Pearson RM Adams MJ Northstone K Tilling K Smith D. Mental health during the COVID-19 pandemic in two longitudinal UK population cohorts. medRxiv [Preprint]. (2020). 10.1101/2020.06.16.20133116 33228822 PMC7844173

[5] Vindegaard N Eriksen Benros M. COVID-19 pandemic and mental health consequences: systematic review of the current evidence. Brain Behav Immun. (2020) 89:531–42. 10.1016/j.bbi.2020.05.048 32485289 PMC7260522

[6] Racine N Korczak DJ Madigan S. Evidence suggests children are being left behind in COVID-19 mental health research. Eur Child Adolesc Psychiatry. (2020). 10.1007/s00787-020-01672-8. [Epub ahead of print]. 33151431 PMC7643721

[7] Liang L Ren H Cao R Hu Y Qin Z Li C. The effect of COVID-19 on youth mental health. Psychiatr Q. (2020) 91:841–52. 10.1007/s11126-020-09744-3 32319041 PMC7173777

[8] Xie X Xue Q Zhou Y Zhu K Liu Q Zhang J. Mental health status among children in home confinement during the coronavirus disease 2019 outbreak in Hubei Province, China. JAMA Pediatr. (2020) 7:1–3. 10.1001/jamapediatrics.2020.1619 32329784 PMC7182958

[9] Zhou SJ Zhang LG Wang LL Guo ZC Wang JQ Chen JC. Prevalence and socio-demographic correlates of psychological health problems in Chinese adolescents during the outbreak of COVID-19. Eur Child Adolesc Psychiatry. (2020) 29:749–58. 10.1007/s00787-020-01541-4 32363492 PMC7196181

[10] Luijten MAJ van Muilekom MM Teela L van Oers HA Terwee CB Zijlmans J. The impact of lockdown during the COVID-19 pandemic on mental and social health of children and adolescents. Qual Life Res. (2021). 10.1007/s11136-021-02861-x. [Epub ahead of print]. 33991278 PMC8122188

[11] Pinquart M. Health-related quality of life of young people with and without chronic conditions. J Pediatr Psychol. (2020) 45:780–92. 10.1093/jpepsy/jsaa052 32642762

[12] Silva N Pereira M Otto C Ravens-Sieberer U Canavarro MC Bullinger M. Do 8- to 18-year-old children/adolescents with chronic physical health conditions have worse health-related quality of life than their healthy peers? A meta-analysis of studies using the KIDSCREEN questionnaires. Qual Life Res. (2019) 28:1725–50. 10.1007/s11136-019-02189-7 31055778

[13] Varni JW Limbers CA Burwinkle TM. Impaired health-related quality of life in children and adolescents with chronic conditions: a comparative analysis of 10 disease clusters and 33 disease categories/severities utilizing the PedsQL^TM^ 4.0 Generic Core Scales. Health Qual Life Outcomes. (2007) 5:43. 10.1186/1477-7525-5-43 17634123 PMC1964786

[14] Bastiaansen D Koot HM Ferdinand RF Verhulst FC. Quality of life in children with psychiatric disorders: self-, parent, and clinician report. J Am Acad Child Adolesc Psychiatry. (2004) 43:221–30. 10.1097/00004583-200402000-00019 14726730

[15] Bastiaansen D Ferdinand RF Koot HM. Predictors of quality of life in children and adolescents with psychiatric disorders. Child Psychiatry Hum Dev. (2020) 51:104–13. 10.1007/s10578-019-00914-4 31338645 PMC6965339

[16] van Muilekom MM Luijten MAJ van Oers HA Conijn T Maurice-Stam H van Goudoever JB. Pediatric patients report lower Health-Related Quality of Life in daily clinical practice compared to new normative PedsQL TM data. Acta Paediatr. (2021) 1–13. 10.1111/apa.15872 PMC8360011 33838052

[17] Lee J. Mental health effects of school closures during COVID-19. Lancet Child Adolesc Heal. (2020) 4:421. 10.1016/S2352-4642(20)30109-7 32302537 PMC7156240

[18] Liu JJ Bao Y Huang X Shi J Lu L. Mental health considerations for children quarantined because of COVID-19. Lancet Child Adolesc Heal. (2020) 4:347–9. 10.1016/S2352-4642(20)30096-1 32224303 PMC7118598

[19] Fegert JM Vitiello B Plener PL Clemens V. Challenges and burden of the Coronavirus 2019 (COVID-19) pandemic for child and adolescent mental health: a narrative review to highlight clinical and research needs in the acute phase and the long return to normality. Child Adolesc Psychiatry Ment Health. (2020) 14:1–1. 10.1186/s13034-020-00329-3 32419840 PMC7216870

[20] Racine N Hartwick C Collin-Vézina D Madigan S. Telemental health for child trauma treatment during and post-COVID-19: limitations and considerations. Child Abus Negl. (2020) 110:1–6. 10.1016/j.chiabu.2020.104698 32839022 PMC7437482

[21] Mutluer T Doenyas C Aslan Genc H. Behavioral implications of the covid-19 process for autism spectrum disorder, and individuals' comprehension of and reactions to the pandemic conditions. Front Psychiatry. (2020) 11:1. 10.3389/fpsyt.2020.561882 33304279 PMC7701051

[22] Conti E Sgandurra G De Nicola G Biagioni T Boldrini S Bonaventura E. Behavioural and emotional changes during covid-19 lockdown in an italian paediatric population with neurologic and psychiatric disorders. Brain Sci. (2020) 10:1–5. 10.3390/brainsci10120918 33260987 PMC7760933

[23] Bobo E Lin L Acquaviva E Caci H Franc N Gamon L. How do children and adolescents with Attention Deficit Hyperactivity Disorder (ADHD) experience lockdown during the COVID-19 outbreak? Encephale. (2020) 46:S85–92. 10.1016/j.encep.2020.05.011 32522407 PMC7276130

[24] Bruining H Bartels M Polderman TJC Popma A. COVID-19 and child and adolescent psychiatry: an unexpected blessing for part of our population? Eur Child Adolesc Psychiatry. (2020). 10.1007/s00787-020-01578-5. [Epub ahead of print]. 32623697 PMC7335225

[25] van Gorp M Maurice-Stam H Teunissen LC van de Peppel-van der Meer W Huussen M Schouten-van Meeteren A. No increase in psychosocial stress of Dutch children with cancer and their caregivers during the first months of the COVID-19 pandemic. Pediatric Blood Cancer. (2021) 68:e28827. 10.1002/pbc.28827 33251717 PMC7744828

[26] Pinar Senkalfa B Sismanlar Eyuboglu T Aslan AT Ramasli Gursoy T Soysal AS Yapar D. Effect of the COVID-19 pandemic on anxiety among children with cystic fibrosis and their mothers. Pediatr Pulmonol. (2020) 55:2128–34. 10.1002/ppul.24900 32530552 PMC7307121

[27] Tso WWY Wong RS Tung KTS Rao N Fu KW Yam JCS. Vulnerability and resilience in children during the COVID-19 pandemic. Eur Child Adolesc Psychiatry. (2020). 10.1007/s00787-020-01680-8. [Epub ahead of print]. 33205284 PMC7671186

[28] Forrest CB Bevans KB Pratiwadi R Moon J Teneralli RE Minton JM. Development of the PROMIS® pediatric global health (PGH-7) measure. Qual Life Res. (2014) 23:1221–31. 10.1007/s11136-013-0581-8 24264804 PMC3966936

[29] DeWalt DA Thissen D Stucky BD Langer MM DeWitt EM Irwin DE. PROMIS pediatric peer relationships scale: development of a peer relationships item bank as part of social health measurement. Heal Psychol. (2013) 32:1093–103. 10.1037/a0032670 23772887 PMC3865609

[30] Irwin DE Stucky B Langer MM Thissen D Dewitt EM Lai JS. An item response analysis of the pediatric PROMIS anxiety and depressive symptoms scales. Qual Life Res. (2010) 19:595–607. 10.1007/s11136-010-9619-3 20213516 PMC3158603

[31] Irwin DE Stucky BD Langer MM Thissen D DeWitt EM Lai J-S. PROMIS pediatric anger scale: an item response theory analysis. Qual Life Res. (2012) 21:697–706. 10.1007/s11136-011-9969-5 21785833 PMC3245753

[32] Forrest CB Meltzer LJ Marcus CL De La Motte A Kratchman A Buysse DJ. Development and validation of the PROMIS pediatric sleep disturbance and sleep-related impairment item banks. Sleep. (2018) 41:1–3. 10.1093/sleep/zsy054 29546286

[33] Cella D Gershon R Lai J-S Choi S. The future of outcomes measurement: Item banking, tailored short-forms, and computerized adaptive assessment. Qual Life Res. (2007) 16:133–41. 10.1007/s11136-007-9204-6 17401637

[34] Michel G Bisegger C Fuhr DC Abel T. Age and gender differences in health-related quality of life of children and adolescents in Europe: a multilevel analysis. Qual Life Res. (2009) 18:1147–57. 10.1007/s11136-009-9538-3 19774493

[35] Houben-van Herten M Bai G Hafkamp E Landgraf JM Raat H. Determinants of health-related quality of life in school-aged children: a general population study in the Netherlands. PLoS ONE. (2015) 10:1–3. 10.1371/journal.pone.0125083 25933361 PMC4416795

[36] Luijten M van Litsenburg R Terwee CB Grootenhuis MA Haverman L. Psychometric properties of the Patient-Reported Outcomes Measurement Information System (PROMIS®) pediatric item bank peer relationships in the Dutch general population. Qual Life Res. (2021) 30:2061–70. 10.1007/s11136-021-02781-w 33606180 PMC8233291

[37] Klaufus LH Luijten M Verlinden E van der Wal MF Haverman L Cuijpers P. Psychometric properties of the Dutch-Flemish PROMIS® pediatric item banks Anxiety and Depressive Symptoms in a general population. Qual Life Res. (2021). 10.1007/s11136-021-02852-y. [Epub ahead of print]. 33983618

[38] Braun V Clarke V. Using thematic analysis in psychology. Qual Res Psychol. (2006) 3:77–101. 10.1191/1478088706qp063oa

[39] Fazel M Hoagwood K Stephan S Ford T. Mental health interventions in schools in high-income countries. The Lancet Psychiatry. (2014) 1:377–87. 10.1016/S2215-0366(14)70312-8 26114092 PMC4477835

[40] Anthes E. Pocket psychiatry: mobile mental-health apps have exploded onto the market, but few have been thoroughly tested. Nature. (2016) 532:20–3. 10.1038/532020a 27078548

[41] Holmes EA O'Connor RC Perry VH Tracey I Wessely S Arseneault L. Multidisciplinary research priorities for the COVID-19 pandemic: a call for action for mental health science. Lancet Psychiatry. (2020) 7:547–60. 10.1016/S2215-0366(20)30168-1 32304649 PMC7159850

